# Increased carotid IMT in overweight and obese women affected by Hashimoto's thyroiditis: an adiposity and autoimmune linkage?

**DOI:** 10.1186/1471-2261-10-22

**Published:** 2010-05-28

**Authors:** Marco M Ciccone, Giovanni De Pergola, Maria T Porcelli, Pietro Scicchitano, Pasquale Caldarola, Massimo Iacoviello, Guida Pietro, Francesco Giorgino, Stefano Favale

**Affiliations:** 1 Section of Cardiovascular Disease, Department of Emergency and Organ Transplantation, University of Bari, School of Medicine, Bari, Italy; 2 Section of Internal Medicine, Endocrinology, Andrology and Metabolic Disease, Department of Emergency and Organ Transplantation, University of Bari, School of Medicine, Bari, Italy; 3 Cardiology Unit- M. Sarcone Hospital, Terlizzi, Bari, Italy

## Abstract

**Background:**

Hashimoto's thyroiditis is the most important cause of hypothyroidism. It is a systemic disease that can even affect the cardiovascular system, by accelerating the atherosclerotic process. Aim of this study was to examine whether autoimmune thyroiditis has an effect on the intima-media thickness of the common carotid artery (IMT-CCT), independently of the thyroid function and well-known cardiovascular risk factors. Hashimoto's thyroiditis is a systemic disease. The aim is to examine whether autoimmune thyroiditis and adiposity can effect carotid IMT independently of thyroid hormones and cardiovascular risk factors.

**Methods:**

A total of 104 obese women (BMI ≥ 25.0 kg/m^-2^), with FT3 and FT4 serum levels in the normal range and TSH levels < 4.5 μU/ml, were investigated. None of these patients was taking any kind of drug influencing thyroid function. Measurements were made of the IMT-CCT, BMI, waist circumference, blood pressure levels, as well as fasting TSH, FT3, FT4, anti-thyroid antibodies, insulin, fasting glycemia, triglycerides, total and HDL-cholesterol serum concentrations.

**Results:**

Of the 104 women, 30 (28.8%) were affected by autoimmune thyroiditis. Significantly higher values of IMT-CCT (p < 0.05), TSH (p < 0.05), and triglycerides (p < 0.05) were obtained, and significantly lower values of FT4 (p < 0.05), in patients with Hashimoto's thyroiditis as compared to those with a normal thyroid function. When examining the whole group together, at multiple regression analysis Hashimoto's thyroiditis maintained a positive association with the IMT (p < 0.001), independently of age, hypertension, BMI, and the fasting serum levels of TSH, FT3, FT4, insulin, fasting glycemia, triglycerides, total and HDL-cholesterol levels.

**Conclusions:**

The present study shows that Hashimoto's thyroiditis is associated to an increased IMT only in overweight and obese, independently of the thyroid function, BMI and cardiovascular risk factors. These results suggest that Hashimoto's thyroiditis is a marker of evolution of the atherosclerosis if combined to adiposity.

## Background

Although published data [1-3] clearly indicate that there is a close relationship between even sub-clinical thyroid dysfunction and an increased cardiovascular risk, the underlying pathogenic process is still unknown. Researchers have so far concentrated on demonstrating the existence of the correlation and evaluating the extent to which different degrees of thyroid dysfunction increase the risk [4-6], but the study populations have rarely been subdivided on the basis of the type of thyroid disease. We believe that this second approach could highlight the close relationships between the two organs, allowing us to study the natural pathogenic mechanisms underlying the impact of thyroid dysfunction on cardiovascular risk, rather than the effects of the secreted hormones on the cardiovascular system in general [7].

Aim of this study was therefore to investigate whether autoimmune Hashimoto's thyroiditis increases the intima-media thickness of the common carotid artery (IMT-CCA), regardless of thyroid dysfunction or traditional cardiovascular risk factors.

## Methods

### Subjects

A total of 104 overweight or obese women (BMI ≥ 25.0 kg/m^2^) attending the Obesity Clinic of the Institute of Internal Medicine, Endocrinology and Metabolic Disease, and the Emergency and Organ Transplant Department of Bari University General Hospital, were enrolled in the study, inclusion criteria being normal blood FT3 and FT4 concentrations, and TSH levels of <4.5 μU/ml.

Exclusion criteria were smoking habit, thyroid or other known endocrine diseases, cardiovascular diseases (coronary heart disease, arrhythmia, heart failure), vascular brain diseases (stroke or a transient ischemic attack), peripheral obstructive artery disease (claudicatio intermittens, delayed or absent peripheral pulses), documented diabetes mellitus, a family or personal history of severe dyslipidemia (triglyceride or total cholesterol levels of >300 mg/dl), chronic liver disease, known kidney disease or any other chronic severe disease. Women taking any kind of medication other than anti-hypertensive drugs, including β-blockers, were also excluded.

Of the 104 enrolled subjects, 30 had Hashimoto's thyroiditis (serum anti-thyroglobulin [Tg-Ab] and anti-thyroid peroxidase antibodies [TPO-Ab]; the remaining 74 did not have positive serum auto-antibodies and were therefore considered free of autoimmune thyroiditis: these were taken as the controls. All patients had serum fasting glucose levels of <126 mg/dl.

High blood pressure was present in 30% of the women with, and 31% of those without autoimmune thyroiditis. Arterial hypertension was diagnosed on the basis of pre-existing treatment with antihypertensive drugs or the criteria of the Third Report of the National Cholesterol Education Program (NCEP) Expert Panel on Detection, Evaluation, and Treatment of High Blood Cholesterol in Adults (Adult Treatment Panel III) Final Report [8]: i.e. systolic (SBP) and diastolic blood pressure (DBP) levels of ≥130 and ≥85 mmHg. Blood pressure was evaluated using a mercury sphygmomanometer with an appropriate-sized cuff with the patients in sitting position, and the average of three measurements was recorded.

Patients were instructed not to change their dietary habits or practice more sport during the study period.

All patients and controls underwent to an ultrasound assessment of their thyroid morphologies.

The study was approved by the Institutional Review Board of Bari University General Hospital and carried out in accordance with the principles of the Helsinki Declaration; all patients gave informed consent before entering the study.

### Anthropometric evaluations

The following anthropometric parameters were evaluated: weight (kg), height (cm), body mass index (BMI) expressed as weight in kilograms divided by the square of height in metres (normal < 24.9 kg/m^2^; overweight 25-29.9 kg/m^2^; obese ≥ 30 kg/m^2^), and waist circumference (cm).

### Biochemistry analyses

Blood samples were taken fasting at 8.00 a.m., and used to measure fasting glycemia (mg/dl), serum insulin (μUI/ml; normal values [n.v.] 4.3-19.9 μU/ml), FT3 (pg/ml; n.v. 2.2-4.2 pg/ml), FT4 (pg/ml; n.v. 8.1-17.1 pg/ml), TSH (μUI/ml; n.v. 0.3-3.6 μU/ml), total cholesterol (mg/dl), HDL cholesterol (mg/dl), triglycerides (mg/dl), Tg-Ab (UI/ml; n.v. < 50 UI/ml) and TPO-Ab (UI/ml; n.v. < 10 UI/ml). Blood glucose was measured using the glucose-oxidase method (Sclavo, Siena, Italy); serum insulin by radio-immune assay (Behring, Scoppito, Italy); FT3, FT4 and TSH using a competitive photometric method based on the solid phase antigen-linked technique (LIASON FT3, LIASON FT4, LIASON TSH, DiaSorin, Saluggi, Italy); total and HDL cholesterol, and triglyceride levels using an enzymatic method (Boehringer Mannheim, Diagnostica Mannheim, Mannheim, Germany); auto-antibodies by radio-immune assays (Tg-Ab IRMA, Biocode, Sclessin, Belgium; TPO-Ab RIA, Sorin Biomedica, Saluggia, Italy).

### IMT-CCA ultrasonography

All patients underwent a two-dimensional echo-colour Doppler examination of the carotid arteries using a high-definition vascular echograph (Philips Sonos 5500 Ultrasound Scanner) and a 7.5 MHz linear electronic probe. They were placed in supine position with their neck in extension and rolled contralaterally by about 45°. The common carotid arteries were examined in the long axis view using a lateral projection and 2.5× image enlargement; bulbar dilation was considered to identify the border between the distal common carotid artery and carotid bulb [9-13]. None of the patients had atherosclerotic plaques, localised lesions ≥2 mm thick, or stenoses.

The images were focused on the intima-media complex of the arterial wall, frozen, and recorded on a videotape. IMT was measured using a digital device (Hewlett Packard, Borthell, Washington) according to Pignoli [9]: i.e. the distance between the principal border of one echogenic line and the principal border of a second, separated by a relatively hypoechoic space ("the double line pattern"). Three measurements were made 1 cm proximally from the carotid bulb on each side, and the average of the six measurements was recorded. Each scan was made by the same investigator, who was blinded to the patients' clinical history and characteristics.

### Statistical analyses

The data are expressed as mean values ± standard deviation except for the category-specific variables, which are expressed in percentages. Between-group differences were analysed using Student's test for independent samples; frequencies were compared using the chi-squared exact test. The correlations between parameters were analysed using Pearson's linear correlation coefficient. The determinants of the independent IMT-CCA variable were assessed by multiple regression analysis. All the analyses were made using STATISTICA 6.1 for Windows software (StatSoft Inc., Tulsa, OK, USA), and p values of < 0.05 were considered statistically significant.

## Results

**Figure 1:**
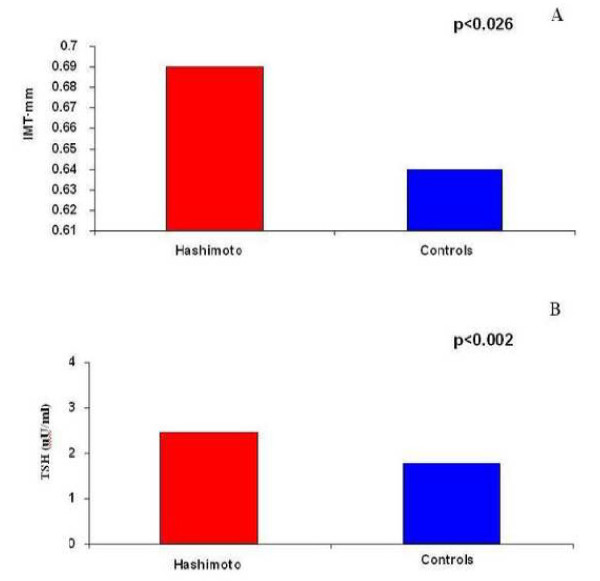
**Panel A: IMT values in Hashimoto patients and controls; Panel B: TSH values in Hashimoto patients and controls**.

Table 1 shows the anthropometric, biochemical and IMT data on the population. There were no significant between-group differences as to demographic and anthropometric variables, but the patients with Hashimoto's thyroiditis had significantly higher IMT (Fig. 1; Panel A), TSH (Fig. 1; Panel B) and triglyceride levels, and significantly lower FT3 levels than the controls.

**Table 1:** Characteristics of the studied population

Variables	*n = 104*	HASHIMOTO *(n = 30)*	CONTROLS *(n = 74)*	*P*
***Age (years)***	39.8 ± 11.1 (18 - 66)	41.1 ± 11.9 (21 - 66)	39.2 ± 10.8 (18 - 61)	0.421
***BMI (kg/m***^***2***^**)**	34.7 ± 6 (25 - 64.6)	34.5 ± 4.6 (27.2 - 45.1)	34.8 ± 6.5 (25 - 64.6)	0.847
***Waist circumference (cm)***	105.9 ± 12.8 (82 - 155)	105.6 ± 10.3 (88 - 132)	106 ± 13.7 (82 - 155)	0.903
***Fasting glycemia (mg/dl)***	92 ± 10.1 (70 - 118)	90.2 ± 9.5 (70 - 109)	92.7 ± 10.3 (70 - 118)	0.253
***Fasting insulinemia (μIU/ml)***	28.4 ± 17.9 (5 - 99)	29.5 ± 16.8 (6 - 72)	28 ± 18.4 (5 - 99)	0.688
***TSH (μU/ml)***	1.96 ± 1.05 (0.38 - 4.5)	2.47 ± 1.17 (0.5 - 4.31)	1.76 ± 0.94 (0.38 - 4.5)	***0.002***
***FT3 (pg/ml)***	3.25 ± 0.38 (2.3 - 4)	3.11 ± 0.4 (2.37 - 3.94)	3.31 ± 0.36 (2.3 - 4)	***0.015***
***FT4 (pg/ml)***	10.29 ± 1.2 (8 - 14.1)	10.26 ± 1.26 (8.44 - 13.3)	10.3 ± 1.19 (8 - 14.1)	0.869
***Total cholesterol (mg/dL)***	194.8 ± 38.3 (133 - 297)	201 ± 43.3 (133 - 297)	192.3 ± 36.1 (134 - 297)	0.299
***HDL cholesterol (mg/dL)***	49.1 ± 10.7 (25 - 85)	49 ± 9.7 (31 - 77)	49.1 ± 11.1 (25 - 85)	0.985
***Triglycerides (mg/dL)***	94.7 ± 43.1 (27 - 248)	114.4 ± 47.4 (47 - 248)	86.7 ± 38.8 (27 - 225)	***0.003***
***IMT (mm)***	0.65 ± 0.11 (0.42 - 0.89)	0.69 ± 0.1 (0.48 - 0.89)	0.64 ± 0.11 (0.42-0.89)	***0.026***
***Systolic blood pressure (mmHg)***	121.7 ± 13.2 (90-160)	120.6 ± 12.1 (105-150)	122.2 ± 13.7 (90-160)	0.573
***Diastolic blood pressure (mmHg)***	79.0 ± 8.6 (65-110)	76.8 ± 6.9 (65-90)	79.8 ± 9.14 (65-110)	0.098
***Hypertension (%)***	30.8	30	31.1	0.915

Mean values ± Standard Deviation (Range)

Subclinical hypothyroidism (normal serum concentrations of FT3 and FT4, and THS ≥ 3.6 μUI/ml) was detected in three of the 74 patients without auto-immune thyroiditis (4.05%) and seven of the 30 patients with Hashimoto' thyroiditis (23.3%).

**Figure 2:**
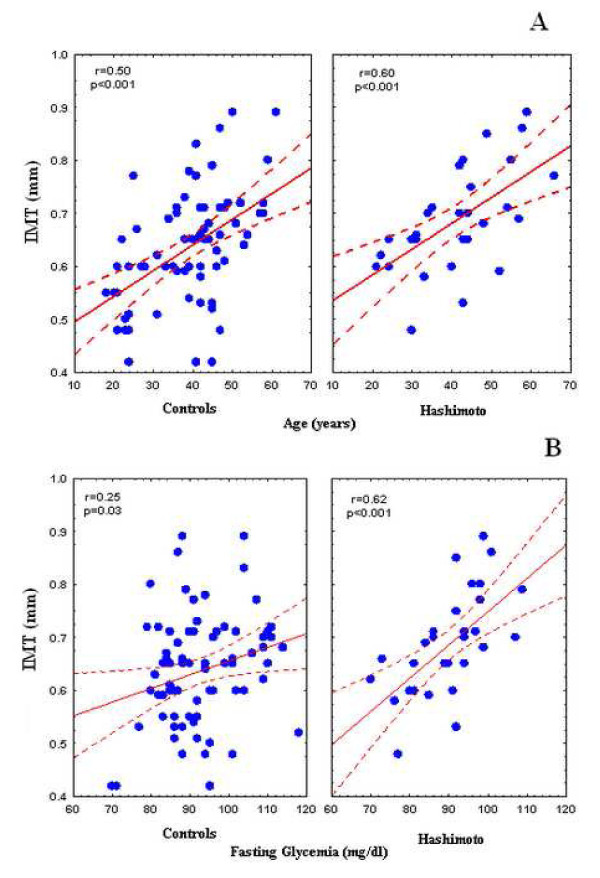
**Panel A: Simple linear correlation of age and IMT in Hashimoto patients and controls; Panel B: Simple linear correlation between fasting glycemia levels and IMT in Hashimoto patients and controls**.

Table 2 shows Pearson's linear correlation coefficients for simple regression analysis of the IMT with the other parameters in the two groups and in the population as a whole. In the population as a whole, IMT was significantly and positively correlated with age, waist circumference, fasting glycemia, total cholesterol, triglycerides and hypertension; in the thyroiditis group, it was correlated with age (Fig. 2; Panel A), fasting glycemia (Fig. 2; Panel B), total cholesterol and triglycerides.

**Table 2:** Pearson's correlation coefficients for simple linear regression between the IMT and all the other parameters in the Hashimoto patients, controls and the population as a whole

	*Hashimoto (n = 30)*	*Controls (n = 74)*	*Total (n = 104)*
***Age***	0.60 *** p < 0.001	0.50 *** p < 0.001	0.53 *** p < 0.001
***BMI***	-0.10 *p = 0.613*	0.10 *p = 0.382*	0.06 *p = 0.572*
***Waist circumference***	0.20 *p = 0.294*	0.22 *p = 0.062*	0.21* p = 0.037
***Fasting glycemia***	0.62 *** p < 0.001	0.25 * p = 0.030	0.31 ** p = 0.001
***Fasting insulinemia***	-0.08 *p = 0.660*	0.05 *p = 0.649*	0.03 *p = 0.779*
***TSH***	0.13 *p = 0.481*	-0.05 *p = 0.693*	0.08 *p = 0.445*
***FT3***	-0.01 *p = 0.951*	0.02 *p = 0.841*	-0.04 *p = 0.694*
***FT4***	0.03 *p = 0.876*	-0.01 *p = 0.941*	0 *p = 0.987*
***Total cholesterol***	0.46 * p = 0.011	0.26 * p = 0.025	0.33 ** p = 0.001
***HDL cholesterol***	0.00 *p = 0.989*	-0.07 *p = 0.570*	-0.05 *p = 0.618*
***Triglycerides***	0.52 ** p = 0.003	0.23 *p = 0.051*	0.36 *** p < 0.001
***Systolic blood pressure***	0.48** P = 0.008	0.34** P = 0.003	0.35*** P < 0.001
***Diastolic blood pressure***	0.21 *P = 0.270*	0.23* P = 0.046	0.18 *P = 0.064*
***Hypertension***	0.23 *p = 0.231*	0.28 * p = 0.016	0.26 ** p = 0.009

* P < 0.05; ** P < 0.01; *** P < 0.001

Table 3 shows the multiple regression analysis coefficients for the population as a whole, with the IMT as the dependent variable and Hashimoto's thyroiditis, age, BMI (Model 1) or waist circumference (Model 2), fasting glycemia, fasting insulin, FT3, FT4, TSH, total cholesterol, HDL-cholesterol, triglycerides and hypertension as independent variables. The two models showed a good fit (R^2 ^= 0.41, P < 0.001 in each case) and the IMT-CCA retained its independent and positive association with age and the reference group (Hashimoto thyroiditis *vs *controls). The explanatory contribution of the other variables was not significant. Moreover, including in the analysis a dichotomic variables to take in account the presence of TSH lower than 3.5, age and Hashimoto remain positively associated with IMT in model 1 (respectively, p < 0.001 and p = 0.048) and model 2 (respectively, p < 0.001 and p = 0.049).

**Table 3:** Model 1-2: Whole population multiple linear regression

	*Model 1*		*Model 2*	
		
	β	*P value*	β	*P value*
***Hashimoto***	0.0454	*0.037*	0.0451	***0.039***
***Age***	0.0045	*0.000*	0.0045	***0.000***
***BMI***	-0.0017	0.372	-	-
***Waist circumference***	-	-	0.0006	0.518
***Glycemia***	0.0019	0.064	0.0015	0.162
***Insulinemia***	-0.0004	0.479	-0.0008	0.224
***TSH***	0.0092	0.313	0.0066	0.471
***FT3***	0.0477	0.061	0.0400	0.111
***FT4***	0.0058	0.437	0.0061	0.410
***Total cCholesterol***	0.0000	0.880	-0.0001	0.832
***HDL cholesterol***	-0.0012	0.194	-0.0011	0.252
***Triglycerides***	0.0002	0.472	0.0002	0.415
***Hypertension***	0.0319	0.138	0.0241	0.254

[IMT-CCA: dependent variable; Hashimoto, age, BMI, fasting glycemia, fasting insulinemia, TSH/FT3/FT4, total/HDL cholesterol, triglycerides, hypertension: independent variables (R^2 ^= 0.41, P < 0.001)]

## Discussion

Hashimoto's thyroiditis is one of the most popular thyroid disease. The diagnosis of Hashimoto's thyroiditis was defined on the basis of the presence of high serum thyroid peroxidase antibody concentrations and of the ultrasound examination, showing a hypoechogenic thyroid. Serum thyroid peroxidase antibodies were considered high whether their concentrations were higher than 40 UI/ml, and they were high in 100 percent of patients with Hashimoto's thyroiditis. Moreover, serum thyroglobulin antibodies were considered high whether their concentrations were higher than 125 UI/ml, and these antibodies were high in less than 50 percent of the patients.

Just the hypoechogenic aspect of thyroid on ultrasound examination let us having a good instrumental parameter in order to detect all patients affected by Hashimoto's thyroiditis

The importance of this study is that Hashimoto' thyroiditis *per se *increases the intima-media thickness of the common carotid artery regardless of thyroid function. Actually, Stamatelopoulos et al. [14] just underlined the positive effects of Hashimoto's thyroiditis on arterial stiffness, regardless the intima-madia thickness. But only overweight and obese women were enrolled in our study and the differences in study group may partly explain the different results between the Stamatelopoulos and our study.

All of the subjects we included in our study had normal serum FT3 and FT4 levels and, although patients with subclinical hypothyroidism were also included, there was no linear correlation between IMT and serum TSH levels. The correlation between IMT and Hashimoto thyroiditis is therefore independent of TSH and thyroid hormone values.

As smoking, diabetes mellitus and severe or familial hyperlipidemia were exclusion criteria [15], and the other atherogenic factors such as abdominal obesity, hypertension, fasting glycemia and insulin levels, and lipidemia typifying the study population did not influence the association between auto-immune thyroiditis and IMT, it can be said that Hashimoto's thyroiditis seems to be an independent cardiovascular risk factor. If this is true, all patients with Hashimoto's thyroiditis (including euthyroid patients) may benefit from L-thyroxin. Besides, Monzani *et al*. have clearly shown that six months of thyroid-replacement therapy in patients with hypothyroidism are sufficient to reduce carotid intima-media thickness [16,17].

The pathogenetic mechanism underlying the increased IMT is not clear, but some hypotheses can be made [18-21]:

1) Although Hashimoto's thyroiditis is related to the IMT regardless of TSH and thyroid hormone levels, we found that our patients with thyroiditis had higher mean serum TSH and triglyceride levels, and lower mean blood FT3 concentrations than the controls. Furthermore, although only a minority of the enrolled women suffered from subclinical hypothyroidism, the percentage was higher among those with auto-immune thyroiditis (23.3% *vs *4.05%). For this reason, a first hypothesis is that Hashimoto thyroiditis, by reducing the thyroid function, is *per se *responsible for increasing the IMT even in subjects with normal FT3 and FT4 levels.

2) As expected, the IMT was directly correlated with age, waist circumference, hypertension, fasting glycemia, and total cholesterol and triglyceride levels, thus confirming the findings of previously published studies [21-23]. The correlation between the IMT and fasting glycemia is particularly interesting because none of the patients had fasting glycemia levels of >110 mg/dl, which suggests a second hypothesis, namely that defective glucose homeostasis may also be *per se *a cardiovascular risk factor in non-diabetic subjects [24-27].

We did not find a significant association between the IMT and fasting insulin levels, although high insulin concentrations are considered a surrogate marker of insulin resistance [15]. However, this is in line with the findings of previous studies that have not demonstrated a clear relationship between insulin concentrations and the angiographically-assessed severity of coronary disease [28,29]. Nevertheless, it is possible that determining fasting insulin concentrations by means of an immuno-assay is only an indirect measure of insulin resistance and cannot be compared with measures of *in vivo *hormone sensitivity indicating an independent association between insulin resistance and the IMT-CCA.

3) Finally, Hashimoto's thyroiditis is an auto-immune disease, and it is well-known that a number of auto-immune diseases are associated with arteritis, accelerated atherosclerosis progression and an increased cardiovascular risk (rheumatoid arthritis, systemic lupus erythematosus, antiphospholipid antibody syndrome...) [30], and so it cannot be excluded that Hashimoto's thyroiditis may in itself be responsible for auto-immune or inflammation-based arteritis. We have no firm evidence supporting such a hypothesis, but it could be an interesting issue to be addressed in future research. Such a consideration come from even from Taddei and al. study [7] in which they demonstrate the endothelial dysfunction coming from Hashimoto's thyroiditis, independently from others cardiovascular risk factors and rather associated with the autoimmune process by itself. For this reason Taddei et al. pointed out the beneficial use of anti-inflammatory drugs in reducing impaired endothelial function in patients with such a disease.

4) Besides, even though TSH levels were higher in the group with Hashimoto's thyroiditis, BMI and waist circumference were not different between thyroiditis and control groups, thus possibly excluding that higher TSH in the thyroiditis group is due to obesity itself.

## Conclusions

In conclusion, our findings show that Hashimoto's thyroiditis is associated with increased arterial wall IMT regardless of thyroid function and well-known cardiovascular risk factors, such as abdominal obesity, hypertension, fasting glycemia, serum insulin and lipid levels. This seems to suggest that auto-immune thyroiditis is in itself a marker of evolution of the atherosclerotic process, particularly in overweight and obese subjects. It seems due to the linkage between the auto-immune process end the endocrinal state consequence to overweight and obesity.

## Abbreviations

IMT: intima-media thickness; IMT-CCA: intima-media thickness of the common carotid artery; BMI: Body mass index; FT3: free triiodothyronine; FT4: free thyroxine; TSH: thyroid-stimulating hormone; Tg-Ab: serum anti-thyroglobulin; TPO-Ab: anti-thyroid peroxidase antibodies; NCEP: National Cholesterol Education Program; SBP: systolic blood pressure; DBP: diastolic blood pressure

## Competing interests

The authors declare that they have no competing interests.

## Authors' contributions

MMC and GDP conceived and designed the study, analysed and interpreted the data, drafted the article and critically reviewed its intellectual content, and finally approved the version to be submitted for publication.  MTP measured and calculated IMT data, reviewed the article's intellectual content, and finally approved the version to be submitted for publication. PS, PC, MI and PG. analysed the data, reviewed the article's intellectual content, and finally approved the version to be submitted for publication.  FG contributed towards designing the study, interpreting the endocrinological data, critically reviewing the article's intellectual content, and finally approving the version to be submitted for publication. SF contributed towards designing the study, interpreting the cardiological data, critically reviewing the article's intellectual content, and finally approving the version to be submitted for publication. All authors read and approved the final manuscript.

## Pre-publication history

The pre-publication history for this paper can be accessed here:

http://www.biomedcentral.com/1471-2261/10/22/prepub
